# Immigration: analysis, trends and outlook on the global research activity

**DOI:** 10.7189/jogh.08.010414

**Published:** 2018-06

**Authors:** Matthias Trost, Eileen M Wanke, Daniela Ohlendorf, Doris Klingelhöfer, Markus Braun, Jan Bauer, David A Groneberg, David Quarcoo, Dörthe Brüggmann

**Affiliations:** 1 Goethe University Frankfurt, Department of Gynecology and Obstetrics, Frankfurt am Main, Germany; 2 Goethe University Frankfurt, Institute of Occupational Medicine, Social Medicine and Environmental Medicine, Frankfurt am Main, Germany; * Equal authors’ contribution

## Abstract

**Background:**

Immigration has a strong impact on the development of health systems, medicine and science worldwide. Therefore, this article provides a descriptive study on the overall research output.

**Methods:**

Utilizing the scientific database Web of Science, data research was performed. The gathered bibliometric data was analyzed using the established platform NewQIS, a benchmarking system to visualize research quantity and quality indices.

**Findings:**

Between 1900 and 2016 a total of 6763 articles on immigration were retrieved and analyzed. 86 different countries participated in the publications. Quantitatively the United States followed by Canada and Spain were prominent regarding the article numbers. On comparing by additionally taking the population size into account, Israel followed by Sweden and Norway showed the highest performance. The main releasing journals are the *Public Health Reports,* the *Journal of Immigrant and Minority Health* and *Social Science & Medicine.* Over the decades, an increasing number of *Public, Environmental & Occupational Health* articles can be recognized which finally forms the mainly used subject area.

**Conclusion:**

Considerably increasing scientific work on immigration cannot only be explained by the general increase of scientific work but is also owed to the latest development with increased mobility, worldwide crises and the need of flight and migration. Especially countries with a good economic situation are highly affected by immigrants and prominent in their publication output on immigration, since the countries’ publication effort is connected with the appointed expenditures for research and development. Remarkable numbers of immigrants throughout Europe compel medical professionals to consider neglected diseases, requires the public health system to restructure itself and finally promotes science.

Immigration has become a vital topic throughout Europe and globally around the world. Effective modes of transportation make it easy to move people quickly around the globe to accept worldwide jobs and boost personal careers [1]. But also new media attracts with transnational information and creates fundamental networks [2]. Additionally conflicts, persecution, human rights violation, or inequality force people to leave their homes and families with the hope to improve their quality of life [1].

In 2015 the number of migrants grew up to 244 million persons worldwide. Mainly originating from middle-income countries nearly two thirds of all international migrants live in Europe and Asia followed by Northern America [1]. Likewise, the number of asylum-seekers was reaching an all-time high with around 2.0 million submitted applications in 2015. 54% of the worldwide refugee population are originating from three countries: the Syrian Arab Republic, Afghanistan and Somalia with the main destinations in Germany, the United States and Sweden [3].

The general health of immigrants and refugees is commonly described as equal to the health of the population in the host country. Even sometimes a ‘healthy-immigrant effect’ is noticed which constitutes that ie, chronic conditions are even fewer in the immigrant group [4–6]. But during the period of acculturation a limited access to local health care systems, including individual and public health services, has a severe impact on the health of immigrants and finally on the health of the nation [7].

As frequently, immigrants do not hold any health insurance coverage, health care services need to be accessible regardless of financial or physical factors. But against the presumption that immigrants use emergency departments more often for routine consultations, this group is actually counted with less emergency department or physician’s consultations [8,9]. Looking at the costs of medical care from a public health perspective, given by an overall good health, the younger age and a fewer utilization of the health care system the health care expenses of immigrants are inferior compared to the native-born population [10].

Even health care professionals experience difficulties in the context of providing health care and public health to immigrants [11]. Language barriers, a different understanding of illness and treatment as well as cultural differences with a lack of trust or an inconsistent medical history are part of those complications. Besides this, traumatic experiences leading to psychological issues need to be addressed [11]. Health care professionals not only need to be aware of rare diseases but also need to address the different groups of immigrants to meet their specific health needs [12]. Immigrants or refugees are susceptible to communicable diseases and can be either the epidemiological originator or adversely affected by a public health emergency. Clear International Health Regulations (IHR) are necessary to provide a comprehensive coverage of infectious diseases [13].

Furthermore, neglected diseases like tuberculosis or parasitic infections may emerge, need to be taken into consideration and may form a major public health concern [14]. After previously declining numbers of tuberculosis during the past, as a result of the demographic development and elevated numbers of immigrants, a significant increase of tuberculosis cases was recognized in various immigration countries in 2015 [15]. The tuberculosis risk among immigrants is increased for several years after migrating to a low-prevalence country [16,17]. This implies a growing challenge for the global control of tuberculosis [18].

Despite numerous studies about several immigration areas can be found, there is no thorough scientometric analysis available. Scientometric studies are an integral part of research evaluation, deliver objective data for budget or funding decisions and provide a vital evidence for an impartial quality assessment [19]. This study maps an overview of the international research activity on immigration. It investigates variations and propensities of scientific development and it illustrates priorities, requirements and opportunities of research.

Although the current refugee movement in Europe requires particular attention, the study was not confined to the peculiar issue of refugees or asylum seekers but was planned to observe the spectrum of immigration as a whole over the last decades and centuries.

Immigration is increasingly important and as one of the most dynamic group immigrants can advance their host country by offering cultural diversity or by leading new pathways in science, medicine and technology [1]. The health of immigrants is a major part to well-being. It enables to work, helps building social networks and promotes integration, whereas on the other hand side integration ultimately leads to better health outcomes [20].

## METHODS

Specific benchmarking systems are being used to evaluate the increasing scientific publication output. Therefore, the *New Quality and Quantity Indices in Science (NewQIS)* platform provides tools for objective scientific evaluation and visualization [21]. Scientific output, semi-qualitative indices and quantities of research activity in particular areas of science can be evaluated and transparently compared considering specific scientometric parameters within a distinct time period.

### Data source

The database *Web of Science Core Collection (WoS)* was used to capture the bibliometric information of the listed articles on immigration. As an international, multidisciplinary tool it offers access to literature of biomedicine and other disciplines [22].

### Search and data processing

In the scientific world, the word ‘migration’ is being used with several meanings. To secure a clear differentiation conclusively the search term ‘immigra*’ was selected to perform a title search on biomedical categories of the *WoS Core Collection* without any chronological restriction. The data was captured in September 2016.

### Evaluation criteria

The number of specific publications was analyzed including the publication year, the country of origin and national or international collaborations. Additionally, journals, articles and subject areas, the publication language as well as the authors and their particular institution were taken into account. Furthermore, the total number of citations, the citation rate [23], and the modified country- and issue-specific h-index [24] were evaluated to assess the awareness of the scientific community. To rectify a possible bias of the low publishing countries, only countries with a threshold of at least 30 articles were taken into consideration while assessing the citation rates.

On additionally considering socioeconomic factors ie, the size of the population and the GDP a further interpretation regarding a countries’ performance is possible. Equally to the citation rates, a minimum of at least 30 articles per country was determined to prevent a distortion of the outcomes.

The h-index is defined by the number n of published articles of an author that have been cited at least n-times each [24]. In this study, it has been applied in a modified way, meaning that it is adapted to countries and only includes the evaluated articles.

### Visualization of findings

Utilizing the *density equalizing mapping projection (DEMP)* [25], a two-dimensional cartographic image with variable proportions can be designed. By scaling the country size adjusted to specific parameters, this technique facilitates a swift overview of the gathered results.

## RESULTS

### General parameters

**Figure 1:**
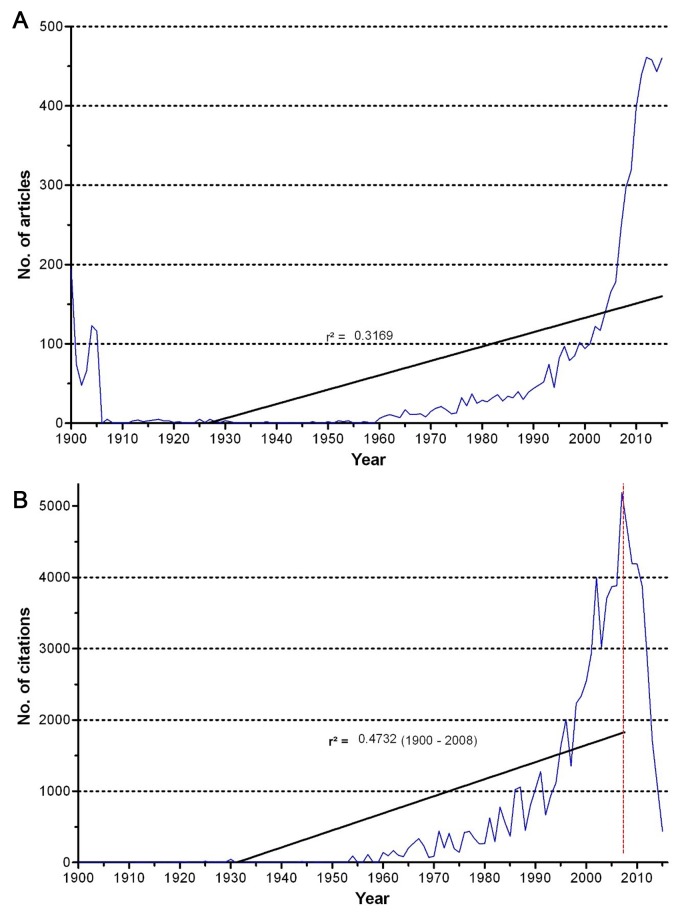
**A.** Chronological development of the total number of publications on immigration, r^2^ = coefficient of determination. **B.** Chronological development of the total number of received citations for articles on immigration, r^2^ = coefficient of determination, dashed line = assumed beginning of the cited half-life effect.

The search delivered a total number of 6763 articles (n). The chronological distribution shows eminent article numbers (n ~ 100/y) in the first years of the 20th century. These articles mainly originate from *Public Health Reports*. The first case report was published in 1902. In 1999, the limit of 100 articles per year was approached. After multiplying the scientific output on immigration an all-time high with n = 461 and n = 460 publications per year was reached in 2012 and 2015 (**Figure 1****, panel A**).

In 1957, first more than a hundred articles on immigration were cited (c = 157) exceeding the limit of one thousand citations first in 1986 (c = 1020). After an excessive increase, the maximum of 5186 citations is seen in 2007. The linear regression of the number of articles over the years showed a coefficient of determination of r^2^ = 0.3169 and respectively for the number of citations of r^2^ = 0.4732 (**Figure 1****, panel B**).

Regarding the top 10 articles on immigration by the number of received citations seven articles are originating from the USA, two articles are from Canada and the most cited article on immigration is published from Japan. The publications were released between 1983 and 2008 and cover several immigration specific topics. A reasonable part of those papers is facing the acculturation and observed health changes after immigration. Apart from cancer, health behaviors and mortality comparisons furthermore obesity, mental disorders or infections are being discussed (**Table 1**).

**Table 1:** Top 10 articles on immigration by number of received citations

Year	Times cited	Title	Author	Country
1991	532	Cancers of the prostate and breast among Japanese and white immigrants in Los Angeles County	Shimizu H et al.	Japan
2002	368	Ethnic-immigrant differentials in health behaviors, morbidity, and cause-specific mortality in the United States: An analysis of two national databases	Singh GK et al.	United States
2003	299	Acculturation and overweight-related behaviors among Hispanic immigrants to the US: the national longitudinal study of adolescent health	Gordon-Larsen P et al.	United States
2006	292	Immigrant youth: Acculturation, identity, and adaptation	Berry JW. et al.	Canada
2004	277	Insights into the 'healthy immigrant effect': health status and health service use of immigrants to Canada	McDonald JT et al.	Canada
2004	276	Obesity among US immigrant subgroups by duration of residence	Goel MS et al.	United States
2008	274	Prevalence of mental illness in immigrant and non-immigrant US Latino groups	Alegria M et al.	United States
1998	266	Adolescent obesity increases significantly in second and third generation US immigrants: The National Longitudinal Study of Adolescent Health	Popkin BM et al.	United States
1983	265	Acquired immune deficiency in Haitians: opportunistic infections in previously healthy Haitian immigrants	Vieira J et al.	United States
2004	258	Immigration and lifetime prevalence of DSM-IV psychiatric disorders among Mexican Americans and non-Hispanic Whites in the United States – Results from the national epidemiologic survey on alcohol and related conditions	Grant BF et al.	United States

### Country specific analysis

**Figure 2:**
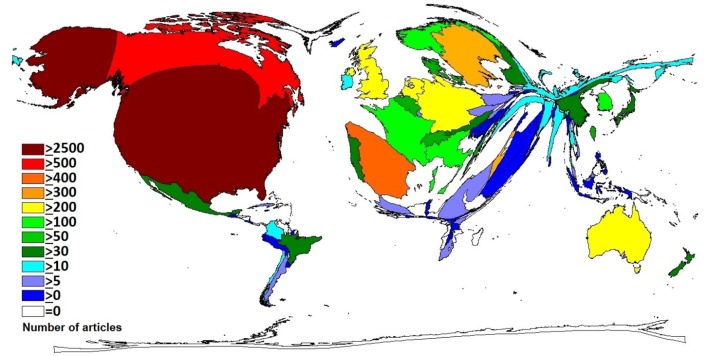
Publishing countries on immigration. DEMP illustrating the number of publications by country.

The identified publications originate from 86 different countries. With a total of n = 2629 articles (38%) the USA reflect around quadruple of the number of articles published in Canada (n = 664, 10%) followed by Spain (n = 454, 7%), Sweden (n = 350, 5%) and Israel (n = 344, 5%) (**Figure 2**).

**Figure 3:**
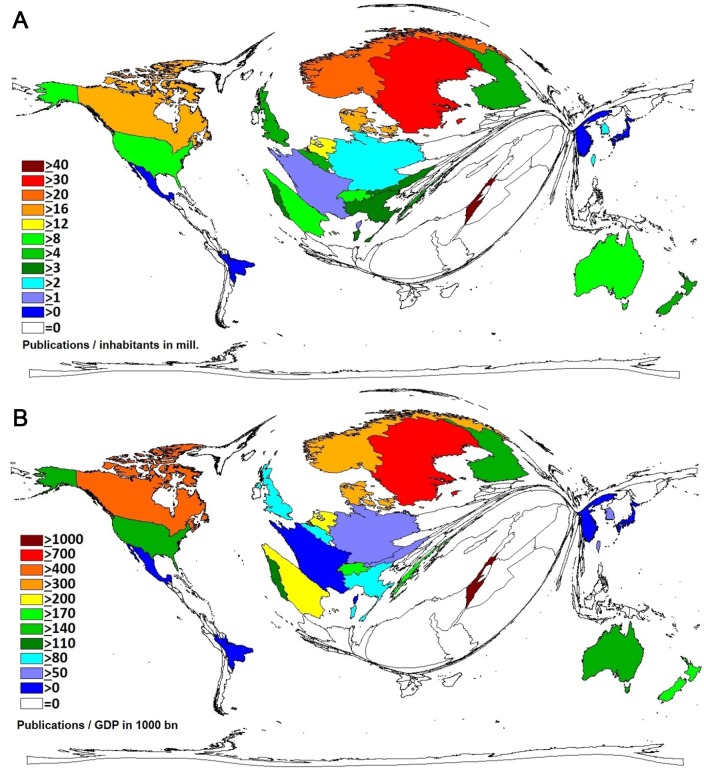
**A.** Countries publishing on immigration by number of publications in relation to the population size in million, threshold: 30 articles per country. **B.** Most publishing countries on immigration by the ratio of publications/GDP in 1000 billion US$, threshold: 30 articles per country.

Setting these outcomes in relation to the size of the particular population of each country results in a different picture (i = publications/inhabitants in million). In this scenario, Israel (i = 42.7) plays the leading role followed by Sweden (i = 35.7), Norway (i = 24.8), Canada (i = 18.9) and Denmark (i = 17.6). The United States (i = 8.2) appear on position ten (**Figure 3****, panel A**).

Reviewing the publication numbers in correlation to the Gross Domestic Product (GDP) in billion (g = publications/GDP in 1000 bn. USD) Israel (g = 1220.3) and Sweden (g = 739.3) are still prominent followed by Canada (g = 406.9), Denmark (g = 378.8) and Norway (g = 362.2). The United States (g = 146.5) appear on position 13 (**Figure 3****, panel B**).

Observing the modified h-index, the United States take the lead again (h = 76). Canada takes the second place (h = 42) and the United Kingdom is on position three (h = 41) followed by Sweden (h = 34) and the Netherlands (h = 33).

Regarding the received citations (c) by country, the order of the top countries is: USA (c = 37 742), Canada (c = 9029), United Kingdom (c = 5766), Sweden (c = 4738), Netherlands (c = 3942).

**Figure 4:**
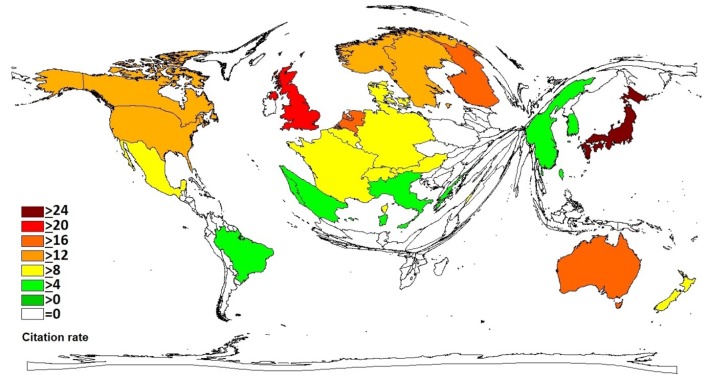
Citation rate for articles on immigration, threshold: 30 articles per country.

Analyzing the citation rate (cr), Japan (cr = 29.3) is taking the lead. The United Kingdom (cr = 20.0) and Finland (cr = 19.4) appear on position two and three followed by the Netherlands (cr = 18.6) and Australia (cr = 16.2). The United States (cr = 14.4) and Canada (cr = 13.6) flag up on positions six and seven directly followed by Sweden (cr = 13.5) (**Figure 4**).

### International collaborations

Observing the scientific partnerships, an interlinked, international network with multi-layered collaborations over national borders can be ascertained.

**Figure 5:**
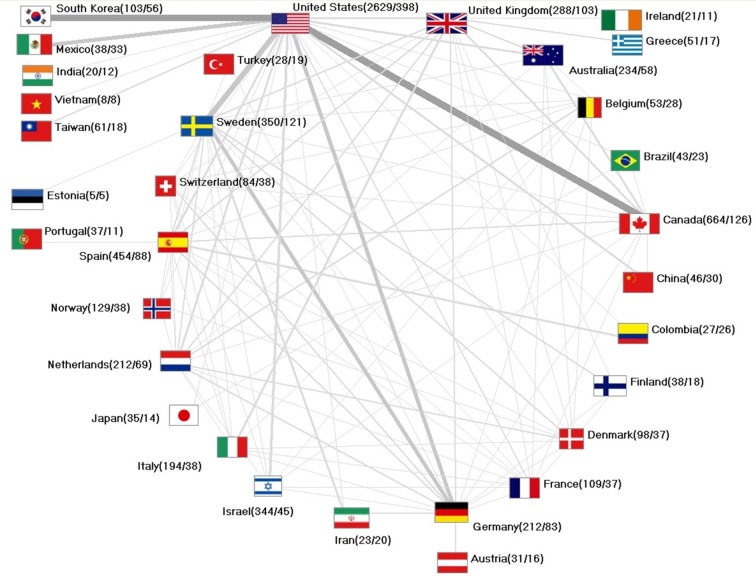
International collaborations. Numbers in brackets: (total number of articles/total number of articles in collaboration).

The highest numbers of direct collaborations between countries were found between the United States and Canada working together on n = 56 papers and the United States together with South Korea counting n = 54 joint publications. The United States and Sweden count n = 49 collaborative papers and Sweden counts in collaboration with Germany n = 33 joint articles. The United States together with Mexico count n = 31 collaborative publications (**Figure 5**).

The United States publish 15% of articles in cooperation. Canada shows 19% of collaborative papers while Sweden represents 35% followed by Germany (39%), South Korea (54%) and Mexico (87%).

### Journals and subject areas

The highest number of immigration papers was published by *Public Health Reports* (n = 638, c = 121). The *Journal of Immigrant and Minority Health* has issued n = 335 papers receiving c = 1671 citations. *Social Science & Medicine* published n = 143 articles and received the highest total amount of citations with c = 4078, whereas the *American Journal of Public Health* received c = 3016 citations with n = 85 articles and the *Hispanic Journal of Behavioral Sciences* gains c = 1288 citations with n = 83 papers (**Table 2**).

**Table 2:** The 15 most cited journals publishing on immigration

Journal Title	Number of articles	Number of citations
Social Science & Medicine (SOC SCI MED)	143	4078
American Journal of Public Health (AM J PUBLIC HEALTH)	85	3016
Journal of Immigrant and Minority Health (J IMMIGR MINOR HEALT)	335	1671
Hispanic Journal of Behavioral Sciences (HISPANIC J BEHAV SCI)	83	1288
Social Psychiatry and Psychiatric Epidemiology (SOC PSYCH PSYCH EPID)	52	1209
LANCET	22	1073
The British Journal of Psychiatry (BRIT J PSYCHIAT)	20	907
British Medical Journal (BRIT MED J)	22	902
Health Affairs (HEALTH AFFAIR)	20	829
Canadian Journal of Public Health (CAN J PUBLIC HEALTH)	50	813
Ethnicity & Health (ETHNIC HEALTH)	54	764
Psychological Medicine (PSYCHOL MED)	16	737
Journal of Epidemiology and Community Health (J EPIDEMIOL COMMUN H)	25	697
The New England Journal of Medicine (N ENGL J MED)	6	670
The American Journal of Psychiatry (AM J PSYCHIAT)	11	668

**Figure 6:**
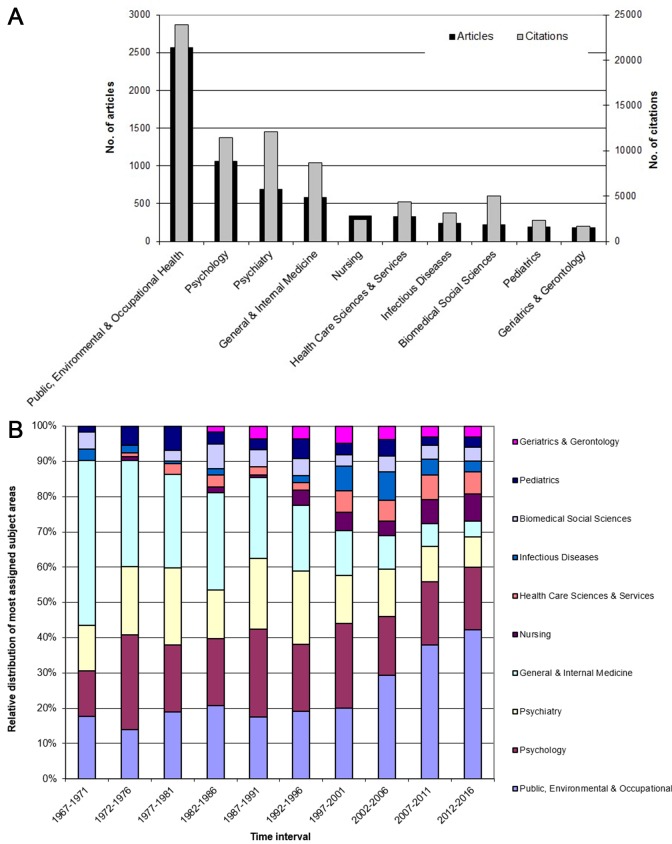
**A.** Most assigned subject areas for papers on immigration by article number and by number of citations. **B.** Relative distribution of the most assigned subject areas by time interval for papers on immigration.

The vast majority of research was found under the subject area *Public, Environmental & Occupational Health* (n = 2564). These articles additionally show the highest citation numbers (c = 23 906). Number two and three form *Psychology* (n = 1065, c = 11 425) and *Psychiatry* (n = 688, c = 12 059) followed by *General & Internal Medicine* (n = 579, c = 8638) and *Nursing* (n = 334, c = 2410) (**Figure 6****, panel A**).

Over the last decades, a change of the applied subject areas can be observed with an increasing trend especially in the field of *Public, Environmental & Occupational Health*. Furthermore, *Psychology* continues to play a major role. Additionally, subject areas like *Nursing* and *Health Care Sciences & Services* gain more visibility (**Figure 6****, panel B**).

## DISCUSSION

The objective of this study was to evaluate the published scientific output using scientometric methods. With the use of the NewQIS System [21] scientific work was assessed regarding quantitative and semi-qualitative parameters considering chronological and geographical factors and additional publication criteria.

This study does not intend an entire content-analysis of the scientific publications on immigration but it considers titles and subject areas. By additionally analyzing scientometric parameters it is possible to investigate characteristics of the worldwide research output as well as the impact on the scientific community [26].

The search term was selected in consensus between the authors. In the light of quality and after thoughtful consideration the expression ‘immigra*’ had to be chosen to acquire a representative data set. With this search we figured out to achieve the best thematic results for the project. The asterisk ‘*’ opens up the option to study ‘immigration’, ‘immigrant’ and other possible spelling variants, keeping in mind that single words like ‘migration’ or ‘emigration’ -if they occur alone- cannot be included in this analysis. But as these terms produced high numbers of artefacts for the purpose of the regarded meaning, it was necessary to exclude these words.

The utilized database was the *Web of Science Core Collection (WoS).* WoS offers search queries through a title or topic selection. The topic search includes the title, the abstract and the author keywords. As an inconsistency can be observed in the structure of keywords [27] and as an abstract search without scanning the keywords is not provided by *WoS* a title search was performed accepting the limitation to receive less but higher quality results.

The comparison of *WoS, Scopus* and *Google Scholar*, which are the only sources for citation analyses yet, results in the detection of different citation numbers for high-profile medical articles [28]. While Scopus covers a wider journal range it has a strong limitation by publishing only recent articles after 1995 [29]. *Google Scholar* lacks in less frequently updated citations [29], consequently *WoS* was the best, but certainly limited, choice.

As *WoS* originates from the United States a bias of the English language has to be taken into detailed consideration. English is used as the universal language of science [30]. This can correspondingly be demonstrated on the gathered results with a clear domination of the English language. As English-language articles reveal a higher impact factor [31] they subsequently receive even more citations.

All parameters based on the amount of citations are affected by incorrect citations or may be manipulated by self-citations [32]. Additionally, several characteristics of citation can be evaluated throughout the different academic disciplines [33].

While assessing the h-index of an author, the age of a scientist has not been taken into account whereas the total period of a scientists’ research activity might influence the results [34]. Furthermore, specific details of an authorship or the country of origin are disregarded.

The citation rate provides single measures but cannot evince the complete work of a researcher. By studying semi-qualitative indicators, the recognition of articles within the scientific community can be investigated. The synopsis of multiple parameters guides to a meaningful statement.

In the first years of the 20th century, remarkable numbers of articles on immigration can be found in *Public Health Reports*. The intention of these reports was to provide epidemiologic information [35]. The first published papers in the early 1900s covered several medical immigration topics. Not only trachoma or hookworms were discussed, noticeable are several articles about ‘insanity’ and ‘mentally defective’ immigrants. It shows that the ethical requirements and the moral attitude during this time were completely different from nowadays.

Looking at the number of articles per annum, increasing scientific work on immigration can be found at the end of the 20th-21st century. On one side, this can be explained by an overall increase of scientific work and publications [36,37]. On the other hand, immigration has become a frequently discussed topic in all areas of life. Not only triggered by worldwide crises and a generally increased mobility [1] immigration has certainly become one of the main topics of public interest in several countries.

The USA releases the vast majority of the total publications. Canada can also be identified as a frequent publishing country. Interestingly Spain, Sweden and Israel are on the next positions which demonstrates the significance of immigration for these countries.

Different countries are equipped with different proportional resources. Not only the national population size but also differing funds for research and development (R&D) need to be taken into consideration [38]. Ranking each countries’ articles in relation to the GDP clearly shows Israel on position one followed by Sweden, Canada and after several mainly European countries the United States on position 13. Comparing the worldwide gross domestic expenditure on R&D (GERD) as a percentage of the GDP Israel and Sweden can correspondingly be found under the top five countries [39]. After adjusting the absolute numbers to the number of inhabitants of each country Israel, Sweden and Norway turn up to be on the first positions which shows their substantial work. These findings clearly validate the importance of immigration for the affected countries.

As scientists are interested in a high visibility and recognition of their work, the preference of collaboration partners within prestigious institutions can explain the collaboration share with the USA. In the scientific world international cooperation is important as it achieves a higher impact [40], it offers access to resources, encourages a spread of knowledge and finally receives more citations [41].

The most cited articles show the broad spectrum of health issues on immigration. Although several subject areas are being used and different authors and institutions are contributing the research, on evaluating the scientific work, a possible country bias needs to be acknowledged as on nine of the top 10 most cited articles the USA are mainly involved. While barely reaching the threshold (n = 30) by publishing 35 articles in total, Japan achieves the highest citation rate. In this context, the bias of one paper from 1991 in collaboration with the USA, on cancer among Japanese US-immigrants, is appreciable. With 532 citations, it is the most cited article in this study indicating the importance of cancer in conjunction with different home countries.

Concerning the subject areas, a difference can be recognized by comparing Italy and Spain with other countries. Relatively these two countries publish a high number of articles using the subject area *Infectious Diseases* with a noticeable focus on tuberculosis. Furthermore, ie, hepatitis, HIV or parasitic diseases are being discussed. This shows the impact of immigration on these countries and the specific health topics they are affected with.

With the general increase of publication numbers interestingly a shift between the topics can be documented over the last years. Although a consistent absolute amount of papers is still being published under the subject area *General & Internal Medicine* the article numbers in the category *Public, Environmental & Occupational Health* are remarkably increasing leading this group to the mainly used subject area. This demonstrates that immigration is not only relevant for individual medicine, but it is in fact an important aspect for public health and subsequently for a whole nation’s health system.

Immigration is a frequently discussed topic which can also be determined by an increase of scientific work about immigration especially during the recent times. During the next years and decades immigration will be in the focus and further scientific work on immigration will be seen. To satisfy this need of science, future international networks as well as interdisciplinary and international scientific cooperation are extremely important.
